# Supplementary material for: Insights into catalysis and regulation of non-canonical ubiquitination and deubiquitination by bacterial deamidase effectors
Source: Nat Commun. 2020 Jun 2;11:2751. doi: 10.1038/s41467-020-16587-w (PMC7265302; doi:10.1038/s41467-020-16587-w)
Supplement: Supplementary file 1 — Supplementary Information [file 41467_2020_16587_MOESM1_ESM.pdf]

Supplementary Information

Insights into catalysis and regulation of non-canonical ubiquitination and deubiquitination by bacterial deamidase effectors

Yong Wang, Qi Zhan, Xinlu Wang, Peipei Li, Songqing Liu, Guangxia Gao, Pu Gao

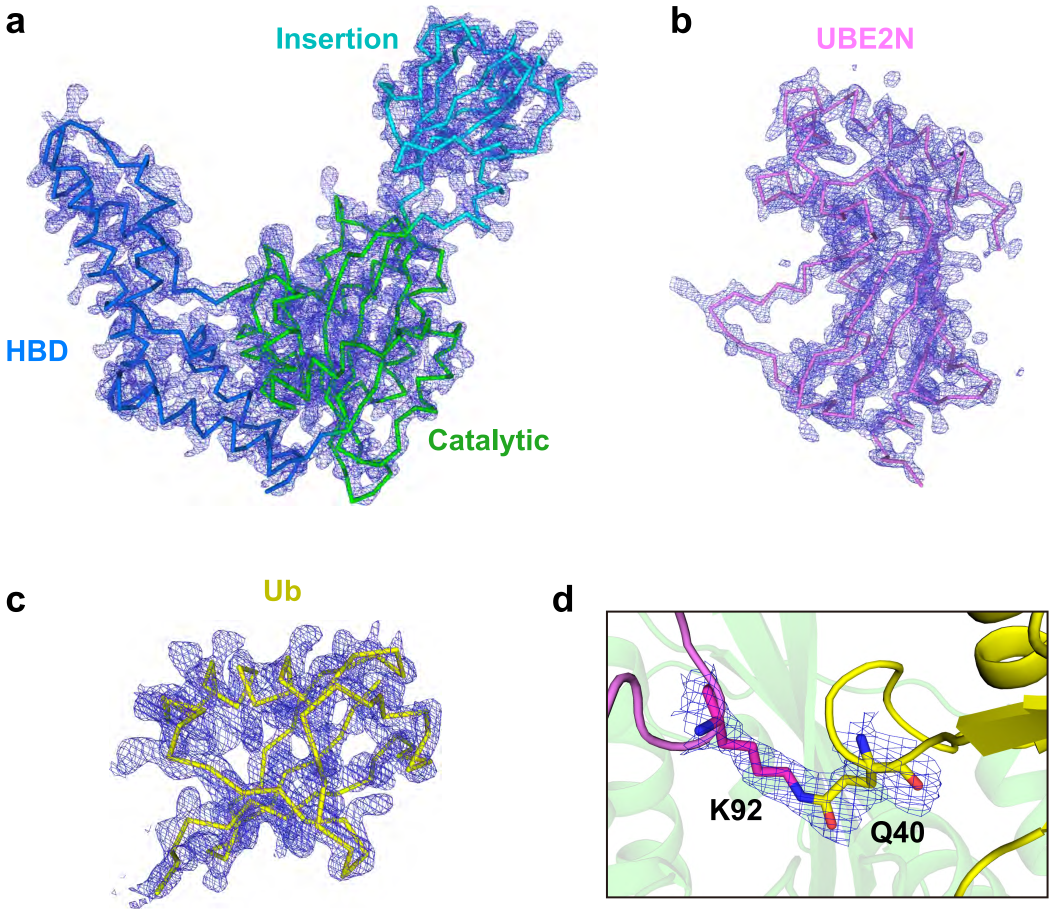

Supplementary Fig. 1: Electron density maps of MavC+UBE2N~Ub complex
a-d, 2Fo-Fc maps contoured at 1.5σ of MavC (a), UBE2N (b), Ub (c), and the isopeptide bond between K92 and Q40 (d).

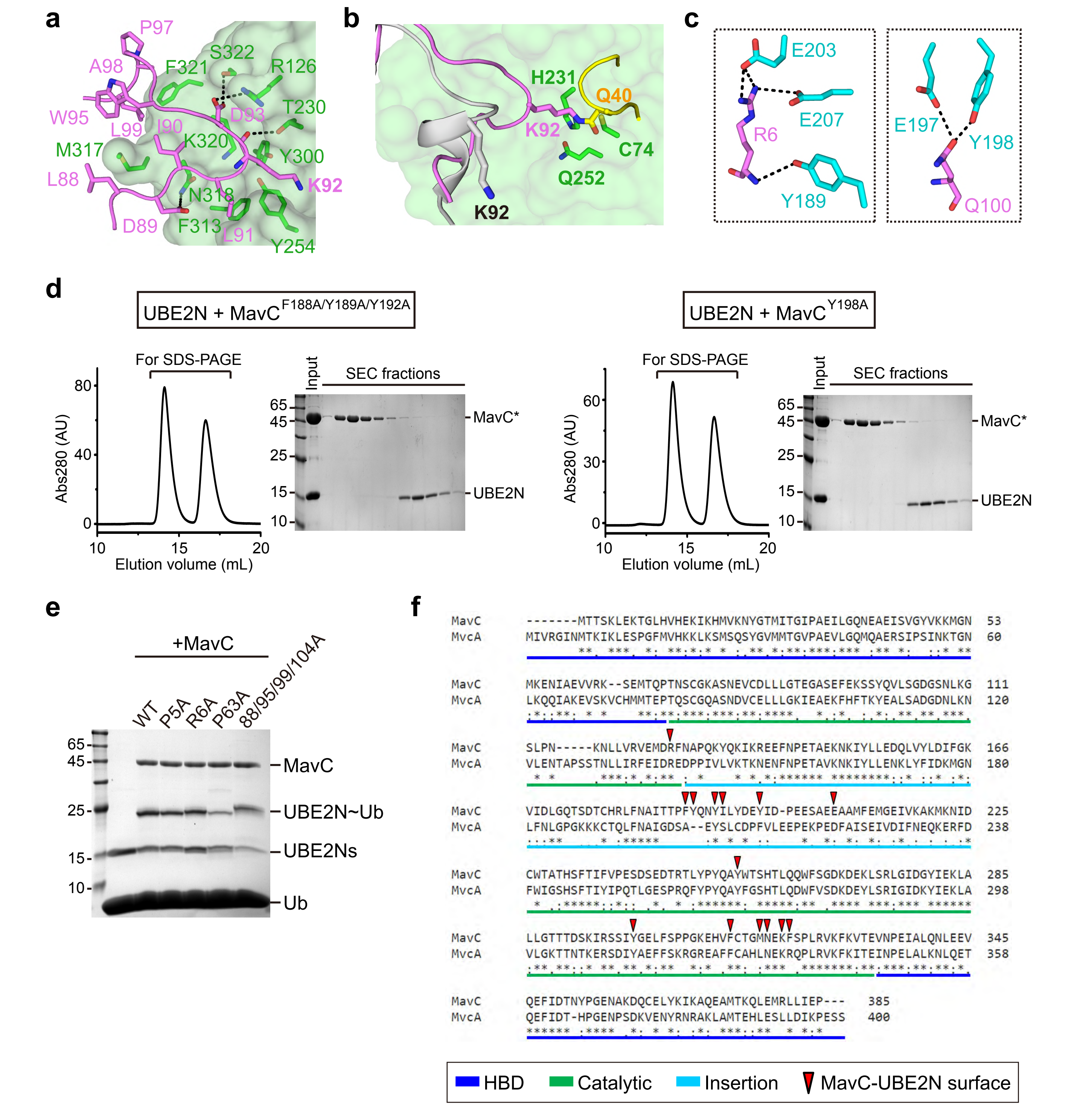

Supplementary Fig. 2: MavC-UBE2N interactions and MavC-MvcA sequence alignment

a, Detailed contacts between the aa 87-100 loop of UBE2N (purple) and the groove of MavC Catalytic domain (green).

b, The aa 87-100 loop of UBE2N (apo state: gray; MavC-bound state: purple) undergoes local conformational changes upon binding to MavC (green), thus positioning K92 to the catalytic pocket of MavC and Q40 of Ub (yellow).

c, Detailed hydrogen bonding interactions between UBE2N (purple) and MavC Insertion domain (cyan).

d, Elution profiles of SEC runs on Superdex 200 10/300 column to test binding of UBE2N with two MavC mutants:F188A/Y189A/Y192A (left) or Y198A (right).

e, Indicated UBE2N proteins were incubated with MavC and Ub. The reaction mixtures were subjected to SDS-PAGE and stained with Coomassie dye.

f, Sequence alignment (by Clustal Omega) between MavC and MvcA. The Catalytic, Insertion, and HBD domains were indicated by green, cyan, and blue lines, respectively. The important residues involved in MavC-UBE2N interactions were indicated by red arrows.

Source data are provided as a Source Data file. Experiments in **d**-**e** were repeated independently three times with similar results.

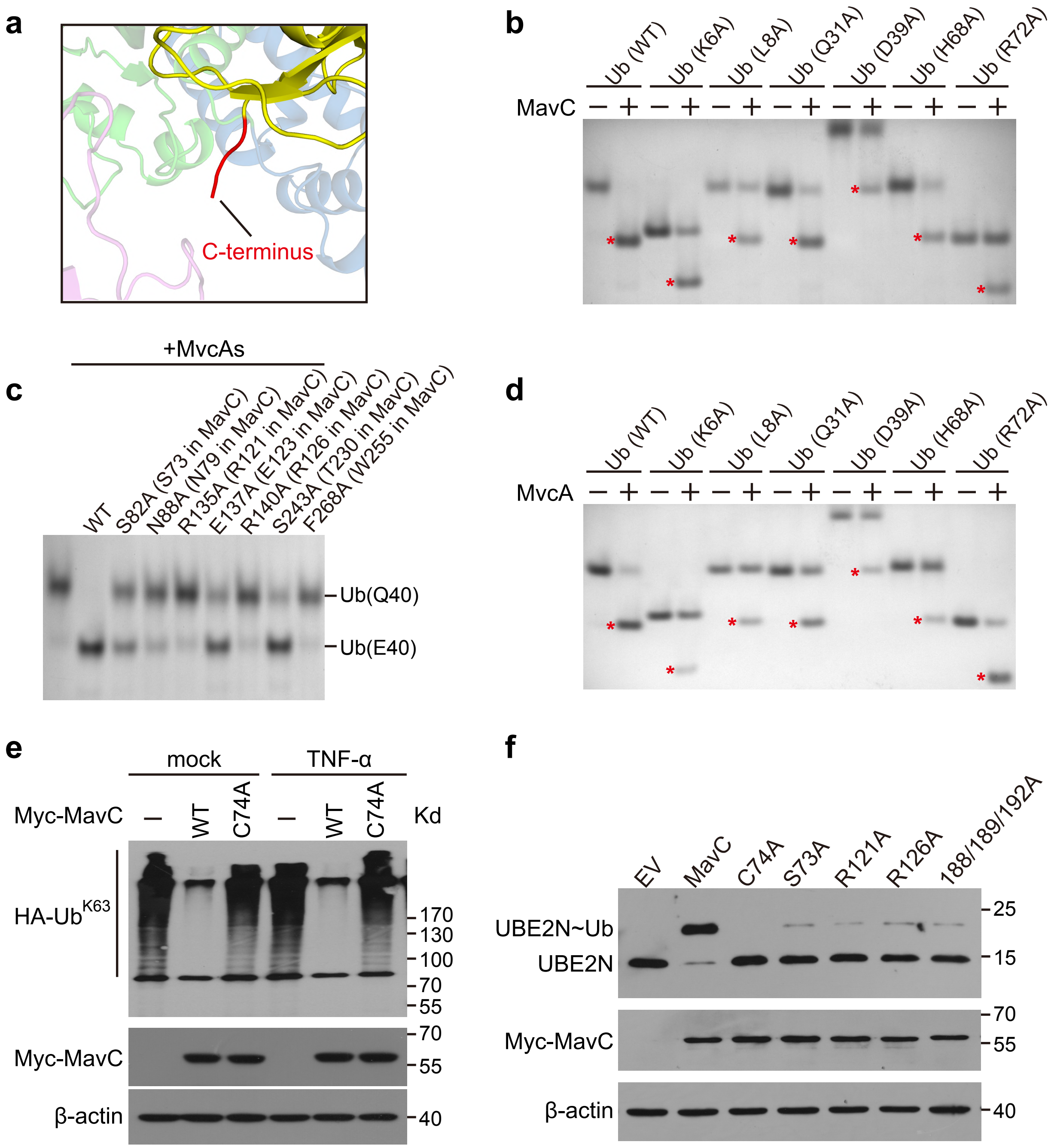

Supplementary Fig. 3: Important roles of MavC/MvcA-Ub interactions and immunoblotting experiments for MavC mutants

a, The C-terminus of Ub (highlighted in red) points into the solvent in the structure of MavC+UBE2N~Ub complex.

b, d, Indicated Ub proteins were incubated with MavC (b) and MvcA (d). The reaction mixtures were subjected to native-PAGE and stained with Coomassie dye. Red asterisks indicate the deamidated Ub bands.

c, In vitro Ub deamidation reactions for various MvcA mutants. The reaction mixtures were subjected to native-PAGE and stained with Coomassie dye.

e, MavC inhibits K63-linked ubiquitination in cells. HEK293T cells transfected with HA-ubiquitin-K63 (all other Lys residues were mutated to Arg except K63) and myc-MavC were mock treated or treated with TNF-α. Cell lysates were subjected to western blotting.

f, Ubiquitination of UBE2N by various MavC mutants in cells. Plasmid encoding MavC or its mutants was transfected into HEK293T cells. The cell lysates were subjected to SDS-PAGE for western blotting. EV: empty vector.

Source data are provided as a Source Data file. Experiments in **b-f** were repeated independently three times with similar results.

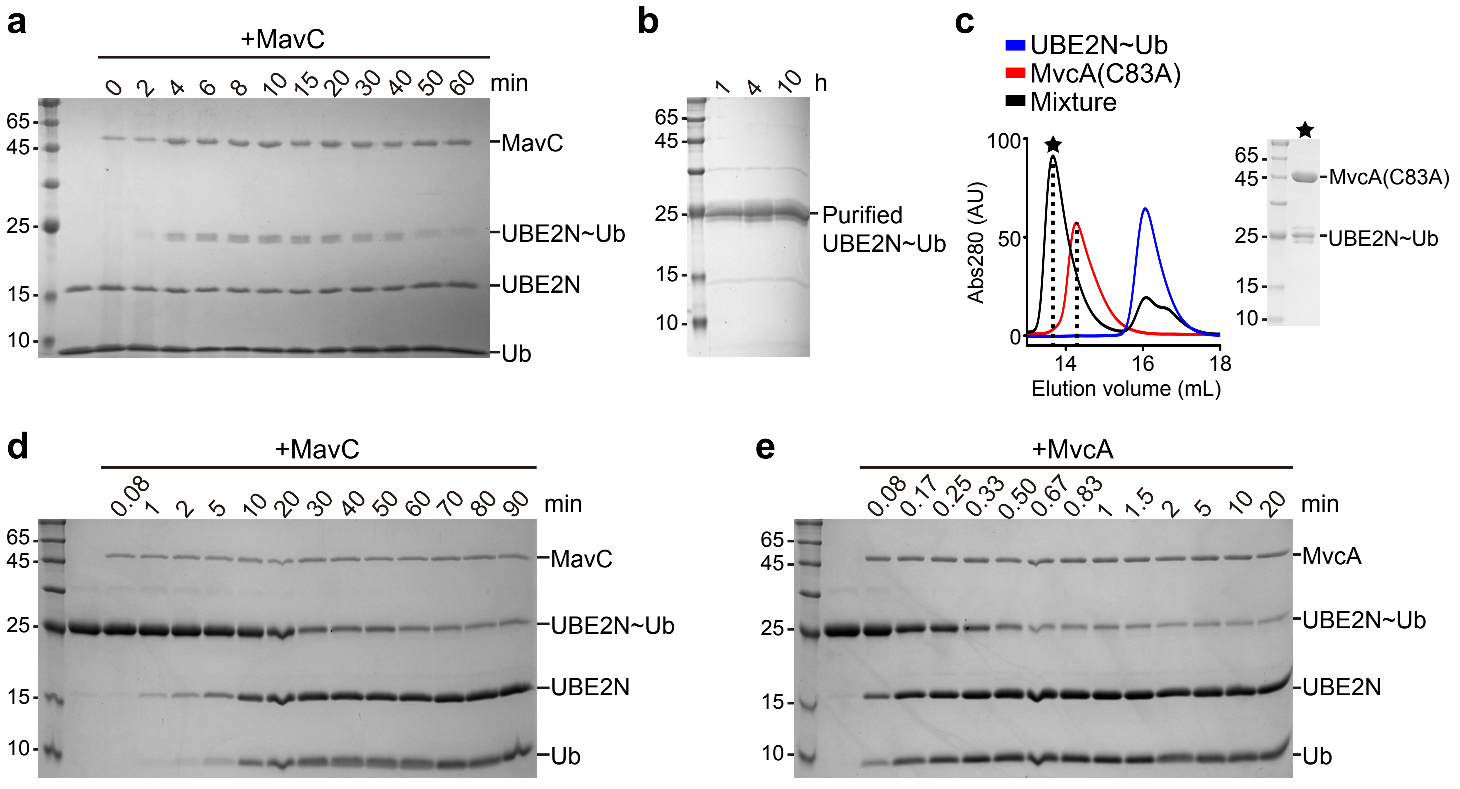

Supplementary Fig. 4: Deubiquitination reactions catalyzed by MavC and MvcA

a, In vitro ubiquitination reactions by incubating MavC with UBE2N and Ub. The reaction mixtures were subjected to SDS-PAGE at different time points. Note that the amount of UBE2N~Ub generated by MavC increased at first and then gradually decreased over time.

b, The purified UBE2N~Ub was incubated in the same buffer and temperature conditions as in (a) and was subjected to SDS-PAGE at indicated time points.

c, Elution profiles of SEC runs on Superdex 200 10/300 column to test binding of UBE2N~Ub with MvcAC83A. Note that MvcAC83A has a considerable shift in elution volume upon binding to UBE2N~Ub. Black asterisk indicates the fraction analyzed by SDS-PAGE.

d, e, In vitro deubiquitination reactions by incubating UBE2N~Ub with same amounts of MavC (d) and MvcA (e). The reaction mixtures were subjected to SDS-PAGE at different time points.

Source data are provided as a Source Data file. Experiments in **a**-**c** were repeated independently three times with similar results. Experiments in **d** and **e** were repeated independently two times with similar results.

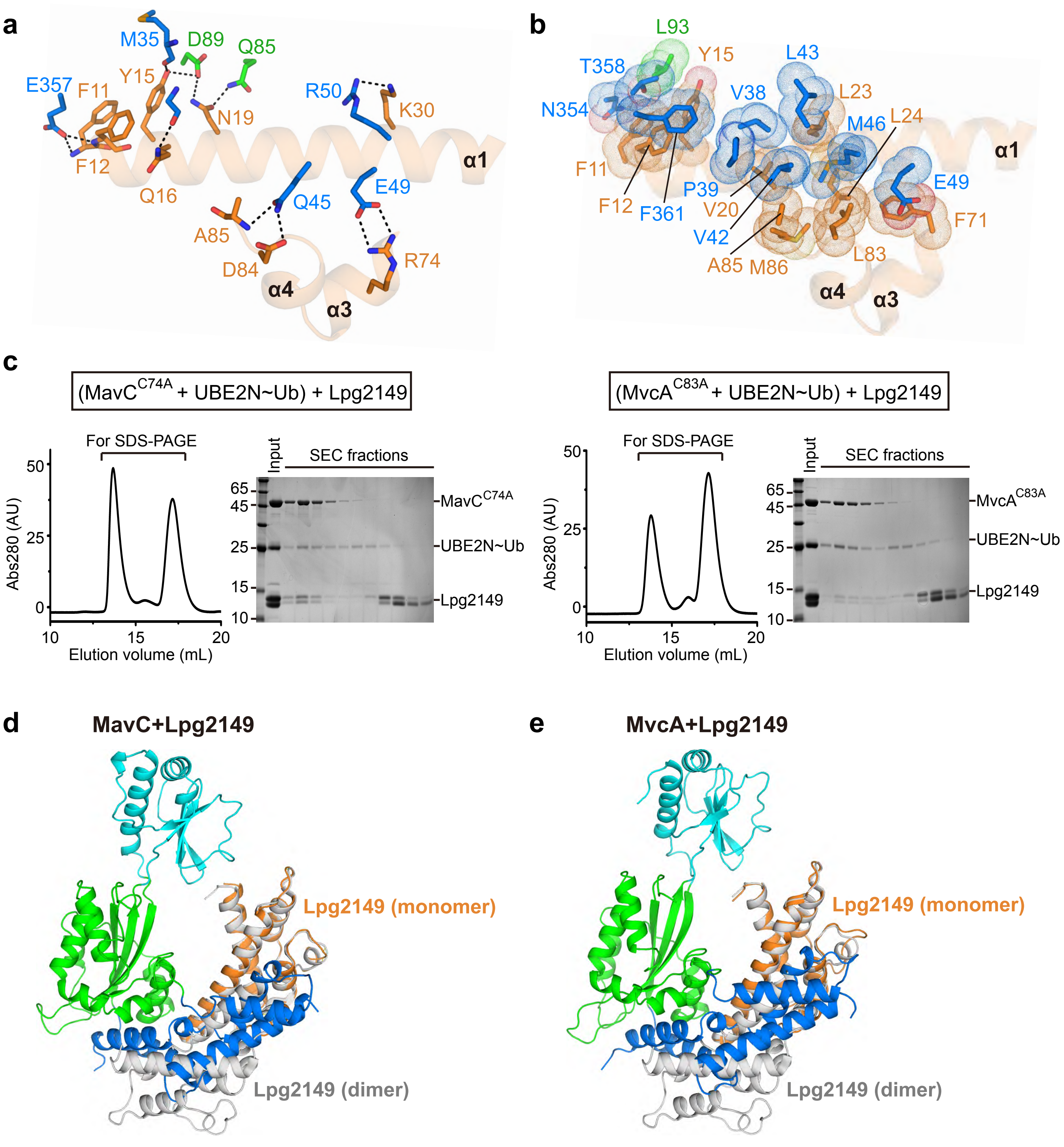

Supplementary Fig. 5: Lpg2149-MvcA interactions and dimer-to-monomer transition of Lpg2149

a, b, Detailed hydrogen bonding (a) and hydrophobic (b) interactions between Lpg2149 and MvcA, with the same color code as in Fig. 5a.

c, Elution profiles of SEC runs on Superdex 200 10/300 column to test the competition binding of Lpg2149 and UBE2N~Ub to MavC^{C74A} (left) or MvcA^{C83A} (right). Note that UBE2N~Ub was first incubated with MavC^{C74A} or MvcA^{C83A} for 30 min, then followed by the addition of Lpg2149.

d, e, Structural superimpositions of the Lpg2149 dimer (gray) with MavC+Lpg2149 (c) and MvcA+Lpg2149 (d) complexes by using one Lpg2149 monomer as the reference point.

Source data are provided as a Source Data file. Experiments in **c** were repeated independently three times with similar results.

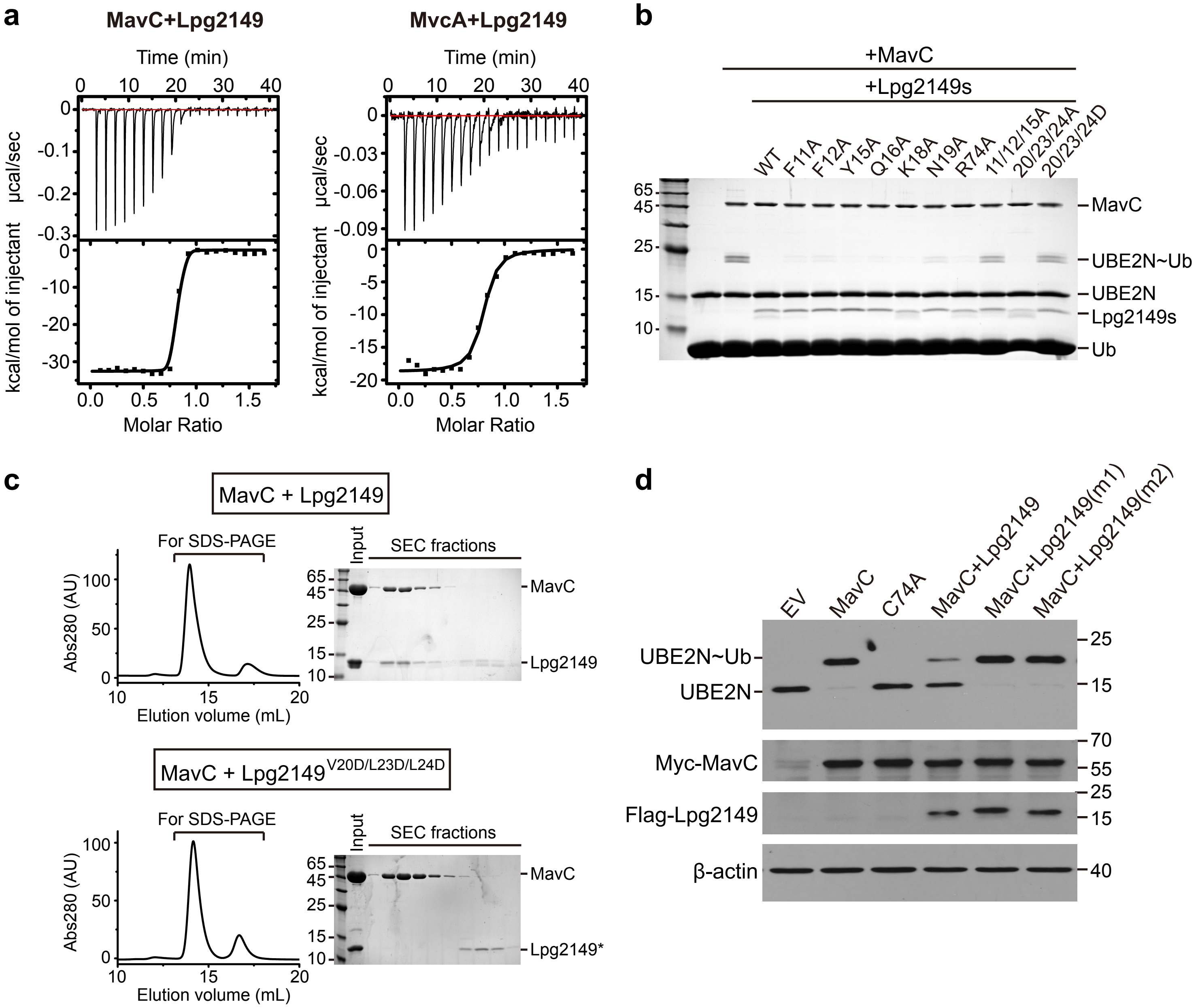

Supplementary Fig. 6: ITC binding between Lpg2149 and MavC/MvcA and inhibition of MavC-mediated ubiquitination by Lpg2149

a, Representative ITC titration results of Lpg2149-MavC (left) and Lpg2149-MvcA (right). The detailed binding parameters were shown in Fig. 6c.

b, Inhibition activities of various Lpg2149 mutants for MavC-mediated UBE2N ubiquitination.

c, Elution profiles of SEC runs on Superdex 200 10/300 column to test binding of MavC with WT Lpg2149 (upper) or its mutant V20D/L23D/L24D (lower).

d, Lpg2149 antagonized MavC-induced UBE2N ubiquitination in cells, and the mutations of Lpg2149 (m1: F11D/F12D/Y15D; m2: V20D/L23D/L24D) decreased the antagonism. Plasmid encoding MavC and Lpg2149 (or its mutants) were co-transfected into HEK293T cells. The cell lysates were subjected to SDS-PAGE for western blotting. EV: empty vector.

Source data are provided as a Source Data file. Experiments in **a-d** were repeated independently three times with similar results.

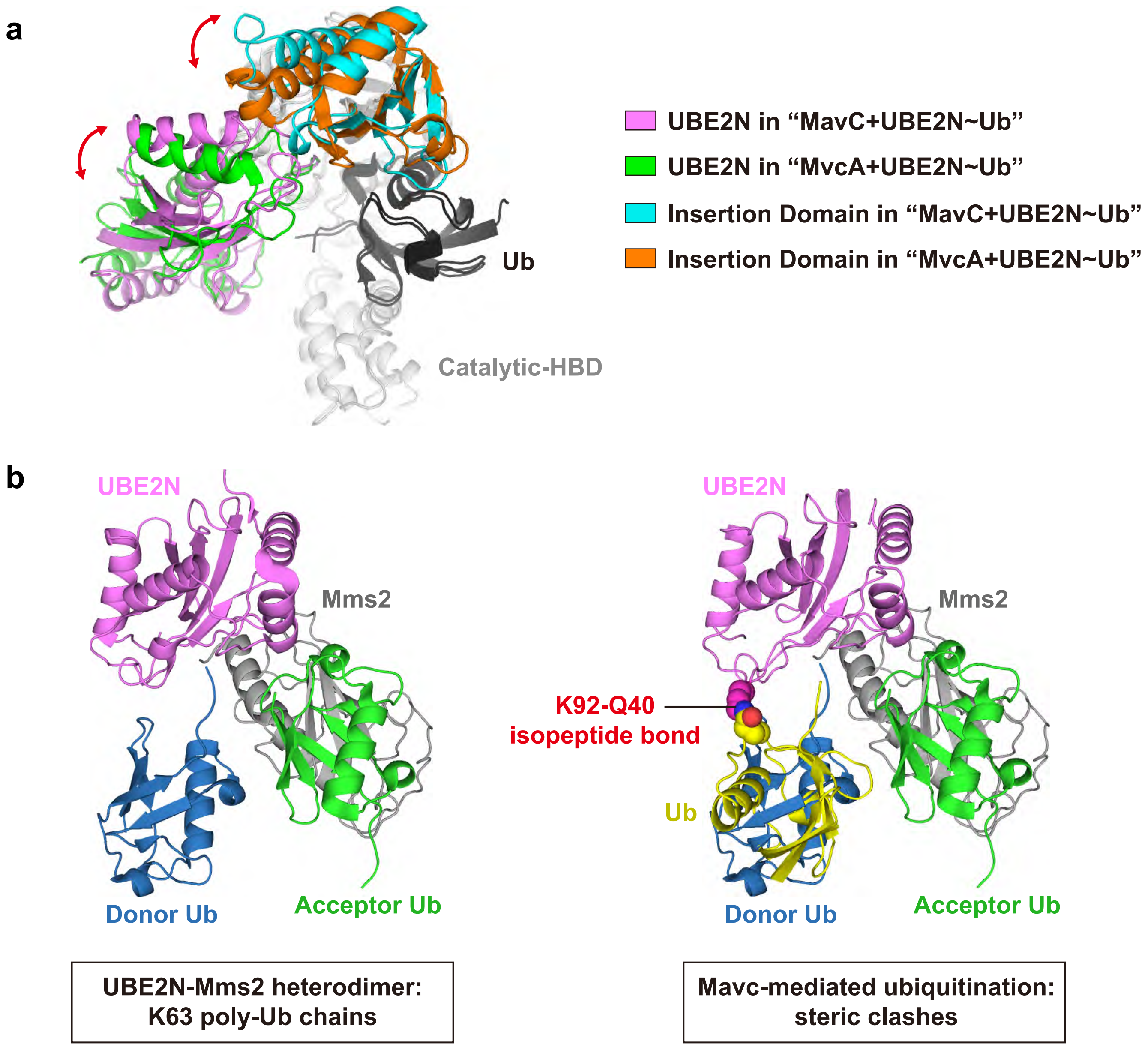

Supplementary Fig. 7: Structural superimpositions of “MavC+UBE2N~Ub vs. MvcA+UBE2N~Ub” and “UBE2N~Ub vs. UBE2N+Mms2+Ub”

a, Structural superimposition between MavC+UBE2N~Ub (current work) and MvcA+UBE2N~Ub (PDB: 6JKY). Note that the Catalytic-HBD regions (gray) and the Ub molecules (black) can be superimposed very well, while the Insertion domains (purple and green) and the UBE2N molecules (cyan and orange) have a considerable rotation between these two structures.

b, Structural superimposition between the conjugated UBE2N~Ub generated by MavC and the UBE2N+Mms2+Ub complex by using UBE2N as the reference point.

Supplementary Table 1 primers used in the study

primer	sequence
F-MavC	TTTTAATCTACAATTAAATATAGGGTATC
R-MavC	TATAAACAGTTCAAGTGACAAATACAC
F-MavC-sumo-B	AGAGAACAGATTGGTGGATCCATGACAACTTCCAAGCTTGA
R-MavC-385-sumo-Not	CGACTTAAGCATTATGCGGCCGCTTACGGCTCTATTAGAAGTCGC
F-MvcA	GAACTGTTTATAATCAAATGATATTGAC
R-MvcA	CAATTTTAGAACAATTTATAATTTAATTG
F-MvcA-sumo-B	AGAGAACAGATTGGTGGATCCATGATTGTGCGTGGCAT
R-MvcA-400-sumo-Not:	CGACTTAAGCATTATGCGGCCGCTTAGCTTGATTCAGGCTTGATG
F-lpg2149	GGGTGTTTGAAAATAACGAAG
R-lpg2149	TGAATGAAGAATTAATATACCAGC
F-lpg2149-sumo-B	AGAGAACAGATTGGTGGATCCATGATGAGTCGCGAAAAAG
R-lpg2149-sumo-Not	CGACTTAAGCATTATGCGGCCGCTTAAGAGTAGTCGGGTGCTTTTTG
F-MavC-C74A	ACAAACAGCGCGGGAAAAGCGAGCAATGAAG
R-MavC-C74A	CGCTTTTCCCGCGCTGTTTGTGGGTTGAGTCA
F-MvcA-C83A	ACTCAGAGTGCGGGCCAAGCCAGTAATGATG
R-MvcA-C83A	GGCTTGGCCCGCACTCTGAGTGGGTTCAGTCATC
F-Ube2N-K94A	TTGAAAGATGCGTGGTCCCCAGCACTGC
R-Ube2N-K94A	TGGGGACCACGCATCTTTCAAAATATCTAAACATATTCTTC
F-hUbi-Q40E	CCCCCCGACGAGCAGAGGCTCATCTTTGCAG
R-hUbi-Q40E	GAGCCTCTGCTCGTCGGGGGGGATGC
F-MvcA-142-sumo-B	AGAGAACAGATTGGTGGATCCGACCCTCCCATTGTATTGG
R-MvcA-239-sumo-Not	CGACTTAAGCATTATGCGGCCGCTTAAAAATCGAATCTCTCTTTTTGTTC
F-MavC-128-sumo-B	AGAGAACAGATTGGTGGATCCAATGCTCCTCAAAAATACCA
R-MavC-225-sumo-Not	CGACTTAAGCATTATGCGGCCGCTTAATCTATGTTTTTCATTTTAGCTTTAAC
F-hUbi-sumo-B	AGAGAACAGATTGGTGGATCCATGCAGATCTTCGTGAAGACC
R-hUbi-sumo-Not	CGACTTAAGCATTATGCGGCCGCTTACCCACCTCTGAGACGCAG
F-lpg2149-9-sumo-B	AGAGAACAGATTGGTGGATCCGGAACTTTTTTTAAAGATTACCAG
R-lpg2149-111-sumo-Not	CGACTTAAGCATTATGCGGCCGCTTAAGTAAAGACAAGTTCTATTTCCCTC
F-2149-F11A	GCTGGAACTGCGTTTAAAGATTACCAGAAAAAAAATG
R-2149-F11A	ATCTTTAAACGCAGTTCCAGCTGCTTTTTCG

F-2149-F12A	GGAACTTTTGCGAAAGATTACCAGAAAAAAAATGTAATG
R-2149-F12A	GTAATCTTTCGCAAAAGTTCCAGCTGCTTTTTC
F-2149-Y15A	TTTAAAGATGCGCAGAAAAAAAATGTAATGAGATTG
R-2149-Y15A	TTTTTTCTGCGCATCTTTAAAAAAAGTTCCAGCTG
F-2149-Q16A	AAAGATTACGCGAAAAAAAATGTAATGAGATTGTTAC
R-2149-Q16A	ATTTTTTTTCGCGTAATCTTTAAAAAAAGTTCCAGC
F-2149-K18A	TACCAGAAAGCGAATGTAATGAGATTGTTACAAGACTC
R-2149-K18A	CATTACATTCGCTTTCTGGTAATCTTTAAAAAAAGTTC
F-2149-N19A	CAGAAAAAAGCGGTAATGAGATTGTTACAAGACTCTCTC
R-2149-N19A	TCTCATTACCGCTTTTTTCTGGTAATCTTTAAAAAAAG
F-2149-R74A	TTTGTTACGCGCCTCTGGCTGGATCCACATAAAG
R-2149-R74A	CAGCCAGAGCGCCGTAACAAAATCAGGAAGTGG
F-2149-F11A/F12A/Y15A	ACTGCGGCGAAAGATGCGCAGAAAAAAAATGTAATGAGATTGTTAC
R-2149-F11A/F12A/Y15A	CTG CGCATCTTTCGCCGCAGTTCCAGCTGCTTTTTCGC
F-2149-V20A/L23A/L24A	AATGCGATGAGAGCGGCGCAAGACTCTCTCGAAAAAATTATCAAC
R-2149-V20A/L23A/L24A	TTGCGCCGCTCTCATCGCATTTTTTTTCTGGTAATCTTTAAAAAAAG
F-2149-V20D/L23D/L24D	AATGACATGAGAGACGACCAAGACTCTCTCGAAAAAATTATCAAC
R-2149-V20D/L23D/L24D	TTGGTCGTCTCTCATGTCATTTTTTTTCTGGTAATCTTTAAAAAAAG
F-hUbc13-P5A	GCCGGGCTGGCGCGCAGGATCATCAAGGAAAC
R-hUbc13-P5A	GATCCTGCGCGCCAGCCCGGCCATGG
F-hUbc13-R6A	GGGCTGCCCGCGAGGATCATCAAGGAAACCC
R-hUbc13-R6A	GATGATCCTCGCGGGCAGCCCGGC
F-hUbc13-P63A	GAAGAATACGCGATGGCAGCCCCTAAAGTAC
R-hUbc13-P63A	GGCTGCCATCGCGTATTCTTCTGGAAGGAATAGTTC
F-hUbc13-L88A/W95A/L99A/V104A	GTCCCCAGCAGCGCAGATCCGCACAGCGCTGCTATCGATCCAGGCC
R-hUbc13 L88A/W95A/L99A/V104A	GCCTTATCTTTCAAAATATCCGCACATATTCTTCCCAACTTGTCTAC
F-hUbi-K6A	ATCTTCGTGGCGACCCTGACCGGCAAG

R-hUbi-K6A	GGTCAGGGTCGCCACGAAGATCTGCATGGATC
F-hUbi-L8A	GTGAAGACCGCGACCGGCAAGACCATCAC
R-hUbi-L8A	CTTGCCGGTCGCGGTCTTCACGAAGATCTGC
F-hUbi-Q31A	GCCAAGATCGCGGATAAAGAAGGCATCCCCC
R-hUbi-Q31A	TTCTTTATCCGCGATCTTGGCCTTCACATTTTC
F-hUbi-D39A	ATCCCCCCCGCGCAGCAGAGGCTCATCTTTG
R-hUbi-D39A	CCTCTGCTGCGCGGGGGGGATGCCTTC
F-hUbi-H68A	GCGCTGGTCCTGCGTCTCAGAG
R-hUbi-H68A	CAGGGTCGACTCTTTCTGG
F-hUbi-R72A	CTGGTCCTGGCACTCAGAGGTGGGTAACTCG
R-hUbi-R72A	ACCTCTGAGTGCCAGGACCAGGTGCAGG
F-hUbi-R74A	CTGCGTCTCGCGGGTGGGTAACTCGAGCAC
R-hUbi-R74A	TTACCCACCCGCGAGACGCAGGACCAGGT
F-MavC-N39A	TTAGGGCAAGCGGAGGCAGAAATTTCGGTTG
R-MavC-N39A	TTCTGCCTCCGCTTGCCCTAATATTTCGGC
F-MavC-E42A	AATGAGGCAGCGATTTCGGTTGGATATGTAAAAAAAATG
R-MavC-E42A	AACCGAAATCGCTGCCTCATTTTGCCCTAATATTTC
F-MavC-E66A	CGAAAATCTGCGATGACTCAACCCACAAACAG
R-MavC-E66A	TTGAGTCATCGCAGATTTTCGAACTACTTCAGC
F-MavC-N72A	CAACCCACAGCGAGCTGCGGAAAAGCGAG
R-MavC-N72A	TCCGCAGCTCGCTGTGGGTTGAGTCATTTCAG
F-MavC-S73A	CCCACAAACGCGTGCGGAAAAGCGAGC
R-MavC-S73A	TTTTCCGCACGCGTTTGTGGGTTGAGTCATTTC
F-MavC-N79A	AAAGCGAGCGCGGAAGTGTGTGATTTACTTTTAGGAAC
R-MavC-N79A	ACACACTTCCGCGCTCGCTTTTCCGCAG
F-MavC-E95A	AGCGAATTCGCGAAATCAAGCTATCAAGTATTATCTGG
R-MavC-E95A	GCTTGATTTCGCGAATTCGCTCGCTCCTTC
F-MavC-R121A	TTACTGGTCGCGGTTGAAATGGATCGATTTAATG
R-MavC-R121A	CATTTCAACCGCGACCAGTAAATTTTTATTGGGTAG
F-MavC-E123A	GTCAGGGTTGCGATGGATCGATTTAATGCTCC
R-MavC-E123A	TCGATCCATCGCAACCCTGACCAGTAAATTTTTATTG
F-MavC-R126A	GAAATGGATGCGTTTAATGCTCCTCAAAAATACC
R-MavC-R126A	AGCATTAAACGCATCCATTTCAACCCTGACC
F-MavC-F188A/Y189A/Y192A	CCTGCGGCGCAAAATGCGATTCTTTATGATGAGTATATTGATCC
R-MavC-F188A/Y189A/Y192A	AATCGCATTTTGCGCCGCAGGGGTGGTAATAGCATTAAATAG
F-MavC-Y198A	TATGATGAGGCGATTGATCCGGAAGAAAGTGC
R-MavC-Y198A	CGGATCAATCGCCTCATCATAAAGAATATAATTTTGATAAAAAG
F-MavC-T230A	TGGACAGCAGCGCATTCTTTTACTATATTTGTTCCTG
R-MavC-T230A	AAAAGAATGCGCTGCTGTCCAACAATCTATGT

F-MavC-Y254A	TACCAGGCAGCGTGGACCAGCCATACTTTGC
R-MavC-Y254A	GCTGGTCCACGCTGCCTGGTAAGGATATAATGTG
F-MavC-W255A	CAGGCATATGCGACCAGCCATACTTTGCAAC
R-MavC-W255A	ATGGCTGGTCGCATATGCCTGGTAAGGATATAATG
F-MavC-Y300A	TCCAGCATTGCGGGAGAACTATTTTCTCCTCCAG
R-MavC-Y300A	TAGTTCTCCCGCAATGCTGGAGCGTATTTTG
F-MavC-F313A	GAACATGTTGCGTGTACCGGGATGAACGA
R-MavC-F313A	CCCGGTACACGCAACATGTTCTTTTCCTGGAG
F-MavC-L36A/I43A	GCGGGGCAAAATGAGGCAGAAGCGTCGGTTGGATATGTAAAAAAATG
R-MavC-L36A/I43A	CGCTTCTGCCTCATTTTGCCCCGC TATTTCGGCAGGAATACCG
F-MavC-N39A/E42A	CAAGCGGAGGCAGCGATTTCGGTTGGATATGTAAAAAAATG
R-MavC-N39A/E42A	AATCGCTGCCTCCGCTTGCCCTAATATTTCGGC
F-MavC-E66A/N72A	ATGACTCAACCCACAGCGAGCTGCGGAAAAGCGAG
R-MavC-E66A/N72A	TGTGGGTTGAGTCATCGCAGATTTTCGAACTACTTCAGC
F-MavC-M317A	TGTACCGGGGCGAACGAAAAATTCTCGCCA
R-MavC-M317A	TTTTTCGTTCGCCCCGGTACAAAAAACATGT
F-hUbc13-sumo-m	GAACAGATTTCGTCGTCGATGGCCGGGCTGCC
R-hUbc13-sumo-m	CCCGGCCATCGACGACGAAATCTGTTCTCTGTGAGCCTC
F-MavC-K320A	ATGAACGAAGCGTTCTCGCCATTACGAGTCA
R-MavC-K320A	TGGCGAGAACGCTTCGTTCATCCCGGTACA
F-MavC-F321A	AACGAAAAAGCGTCGCCATTACGAGTCAAATTTAAAG
R-MavC-F321A	TAATGGCGACGCTTTTTCGTTCATCCCGGTAC
F-MvcA-S82A	CCCACTCAGGCGTGTGGCCAAGCCAGTAATG
R-MvcA-S82A	TTGGCCACACGCCTGAGTGGGTTCAGTCATCATATG
F-MvcA-N88A	CAAGCCAGTGCGGATGTGTGTGAATTGCTTTTAGG
R-MvcA-N88A	ACACACATCCGCACTGGCTTGGCCACAACTC
F-MvcA-R135A	CTATTAATAGCGTTTGAAATTGACAGAGAAGACC
R-MvcA-R135A	AATTTCAAACGCTATTAATAGGTTAGTGCTGGATGG
F-MvcA-E137A	ATACGATTTGCGATTGACAGAGAAGACCCTCCC
R-MvcA-E137A	TCTGTCAATCGCAAATCGTATTAATAGGTTAGTGCTG
F-MvcA-R140A	GAAATTGACGCGGAAGACCCTCCCATTGTATTG
R-MvcA-R140A	AGGGTCTTCCGCGTCAATTTCAAATCGTATTAATAGG
F-MvcA-S243A	TGGATCGGGGCGCATTCTTTTACAATATATATCCCTCA
R-MvcA-S243A	AAAAGAATGCGCCCCGATCCAAAAATCGA
F-MvcA-F268A	CAAGCTTACGCGGGTTCACACACTTTACAGGATTG

R-MvcA-F268A	GTGTGAACCCGCGTAAGCTTGATAAGGATAGAATTGG
F-Ube2N-K92A	GATATTTTGGCGGATAAGTGGTCCCCAGCAC
R-Ube2N-K92A	CCACTTATCCGCCAAAATATCTAAACATATTCTTCCC
F-MavC-Myc-EcoRI	ATGGCCATGGAGGCCCGAATTCGGATGACAACTTCCAAGCTTGA
R-MavC-E-Myc-XhoI	CCCCGCGGCCGCGGTACCTCGAGTTACTTATCACGAAGAACTAACCCG
F-lpg2149-HF-EcoRI	AAAGACGATGACGATAAAGAATTCATGATGAGTCGCGAAAAAG
R-lpg2149-HF-XhoI	CCCCGCGGCCGCGGTACCTCGAGTTAAGAGTAGTCGGGTGCTTTTTG
F-hUbi-HF-EcoRI	AAAGACGATGACGATAAAGAATTCATGCAGATCTTCGTGAAGACC
R-hUbi-HF-XhoI	CCCCGCGGCCGCGGTACCTCGAGTTACCCACCTCTGAGACGCAG
